# Polymer Magnetic Composite Core Based Microcoils and Microtransformers for Very High Frequency Power Applications

**DOI:** 10.3390/mi7040060

**Published:** 2016-04-05

**Authors:** Saravana Guru Mariappan, Ali Moazenzadeh, Ulrike Wallrabe

**Affiliations:** 1 Laboratory for Microactuators, Department of Microsystems Engineering (IMTEK), University of Freiburg, Georges-Koehler-Allee 102, 79110 Freiburg, Germany; guru.mariappan@voxalytic.com (S.G.M.); ali.moazenzadeh@voxalytic.com (A.M.); 2 Voxalytic GmbH, Rosengarten 3, 76228 Karlsruhe, Germany

**Keywords:** microtransformer, polymer magnetic composite (PMC), microcoil, MEMS, wirebonding, very high frequency (VHF), passive components, power conversion, micro-machining

## Abstract

We present a rapid prototyping and a cost effective fabrication process on batch fabricated wafer-level micro inductive components with polymer magnetic composite (PMC) cores. The new PMC cores provide a possibility to bridge the gap between the non-magnetic and magnetic core inductive devices in terms of both the operating frequency and electrical performance. An optimized fabrication process of molding, casting, and demolding which uses teflon for the molding tool is presented. High permeability NiFeZn powder was mixed with Araldite epoxy to form high resistive PMC cores. Cylindrical PMC cores having a footprint of 0.79 mm${}^{2}$ were fabricated with varying percentage of the magnetic powder on FR4 substrates. The core influence on the electrical performance of the inductive elements is discussed. Inductor chips having a solenoidal coil as well as transformer chips with primary and secondary coils wound around each other have been fabricated and evaluated. A core with 65% powder equipped with a solenoid made out of 25 µm thick insulated Au wire having 30 turns, yielded a constant inductance value of 2 µH up to the frequency of 50 MHz and a peak quality factor of 13. A 1:1 transformer with similar PMC core and solenoidal coils having 10 turns yielded a maximum efficiency of 84% and a coupling factor of 96%. In order to protect the solenoids and to increase the mechanical robustness and handling of the chips, a novel process was developed to encapsulate the components with an epoxy based magnetic composite. The effect on the electrical performance through the magnetic composite encapsulation is reported as well.

## 1. Introduction

The ongoing trend towards miniaturization of electronics with proliferation of functionality and performance requires compact and efficient power management at microsystem platforms. The aforementioned aspects are addressed by the power semiconductor industries and various research groups through their ability to deliver advanced processing and functional integration by driving the power management system in the very high frequency regime (VHF, 30 to 300 MHz) [1]. A major challenge is that the further miniaturization of the power modules is limited by the presence of bulky inductive components though the inductance needed at the VHF frequencies are relatively low compared to the systems operating at lower frequencies [2,3].

Typically, inductive components at VHF regime are implemented using bulky air core devices. The size of the inductors can be reduced by integrating soft magnetic cores to achieve a high inductance density, however, at VHF regime the frequency dependent core losses become dominant [4]. Advanced but expensive, multilayer deposition techniques such as electroplating or sputtering are usually adopted to minimize the losses [5,6,7,8]. However, the process complexity remains as an impediment [9,10].

An alternative method is to utilize a composite material comprising metallic magnetic powder separated by a dielectric polymer to increase the inductive device performance for high frequency applications [11]. The dielectric guarantees high electrical resistivity of the core, while the metal grains are too small for significant eddy currents to circulate within the particles, which results in lower losses in the VHF regime. Through careful adjustment of the magnetic permeability on the one hand, and the particle size and shape on the other hand, one can tailor the magnetic response of the core to a specific need. Being a promising technique, it still has various disadvantages for example it is difficult to integrate it into a complementary metal-oxide-semiconductor (CMOS) process since most of the reported fabrication methods involve high temperature sintering and annealing of the magnetic cores [12].

In our approach, these limitations are addressed with the help of an improved epoxy based binder material and a new casting technique integrated with an automated wirebonding process for wafer-level batch fabrication of polymer magnetic composite (PMC) core microcoils and microtransformers. The fabrication of 3D microcoils using an automatic ball-wedge wirebonder was well established in the previous works in our laboratory [13]. So far we fabricated air core and laminated Metglas^®^ 2714 (Metglas Inc., Conway, SC, USA) based magnetic core inductive components [14,15,16]. The proposed extremely versatile methodology in this paper allows us to integrate PMC cores with different magnetic powders and varying cross section, that could result in an inductive component having a moderate inductance density but can be used at the VHF regime with low core losses. This is a straight forward and cost effective approach while it enables reproduction of such magnetic cores in a normal lab space. In this paper, we report a versatile fabrication process to structure cylindrical PMC cores with varying magnetic proportion. The PMC is made out of a high permeability NiFeZn based soft magnetic powder and Araldite based epoxy as the binding dielectric material. The well established wirebonding technique was used to fabricate inductor and transformer chips. The frequency dependent magnetic behavior of the fabricated passive components is compared to the previously reported devices. We also introduce a process for the encapsulation of the fabricated devices with an epoxy based magnetic composite.

## 2. Choice of Core Materials

One of the critical considerations in fabricating PMC cores is the material choice. The polymer binder and the soft magnetic powder determines the viability of the fabrication process and the magnetic behavior of the inductive component, respectively.

### 2.1. Soft Magnetic Powders

The main parameter, often used as a figure of merit for soft magnetic materials, is the relative permeability (${µ}_{r}$), which is a measure for the response of the material to the applied magnetic field. In our case, an appropriate material for the magnetic cores needs to have a high permeability, a high electrical resistivity, and a low coercivity. Pure iron is the most prototypical soft magnetic material. It has a very high saturation flux density and a high permeability but a low resistivity which results in high core losses. However, alloyed iron provides higher magnetic permeability and is expected to provide lower core losses when used as amorphous magnetic ribbon or magnetic powder [17,18], therefore it is a better choice for fabricating composite core devices with high efficiency.

In addition, particle shape and size further influence the eddy current losses and coercivity, thereby, the overall frequency dependent magnetic response of the core [18,19]. These factors depend on the preparation and processing methods of the magnetic alloy powders [20]. Mechanical alloying [21] and borohydride reduction of salt solution containing Fe, Co, and Nd [22] are commonly used for the synthesis of amorphous and partially crystalline magnetic powders.

**Figure 1:**
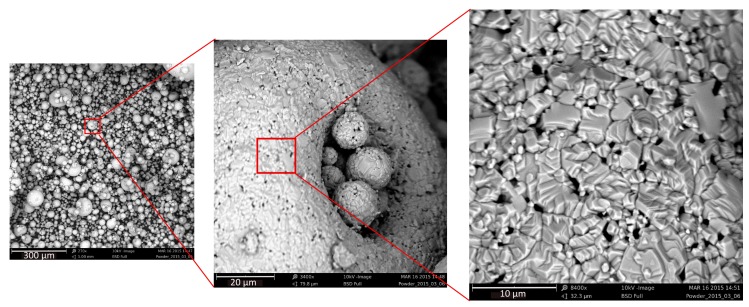
SEM images of NiFeZn soft magnetic powder. Due to the surface tension the particles stick to each other to form a spherical or a toroidal shape.

A brief overview on the available magnetic alloy powders is reported in this section to conclude the choice of a magnetic material. *Fe-Si alloys* are harder and have a higher electrical resistivity than pure iron [23]. For applications requiring a very low hysteresis loss, high permeability, low remanence, and freedom from magnetic aging, *Fe-Si alloys* are an optimal choice. *Fe-Co alloys* have the highest magnetization saturation of all known magnetic alloys [24]. *Fe-Ni alloys* possess the highest permeability of all the soft magnetic alloy [18]. Therefore, these alloys are considered foremost for our application that requires high permeability. In this work, CMD5005, an NiFeZn based soft magnetic powder from the National Magnetics Group was used. The powder is expected to provide a high permeability and a high electrical resistivity at the VHF frequency regime. The properties are listed in Table 1. SEM images of the powder are shown in Figure 1.

### 2.2. Polymer Matrix

One of the main objectives of this work was to insulate the magnetic particles from each other with an insulating dielectric polymer. The binding material should have a low dielectric constant and provide viability for mixing a high percentage of magnetic powder. It should also ensure sufficient adhesion to the substrate to withstand mechanical, thermal, and chemical impacts employed during the fabrication process. In addition, the binder material should have a low viscosity, a high glass transition temperature ($T_{g}$), long pot time (the time it takes to double the initially mixed viscosity), low curing temperature, and a high Young’s modulus. Based on the study of various publications [23,24,26,27,28,29,30,31,32] and intensive search for commercial availability, suitable polymers have been identified which are listed in Table 2.

For our fabrication process, two component epoxies seemed to be a viable option since there are wide varieties of such epoxies with low viscosity and optimal pot time (from 25 to 60 min), hence, they can be processed with a minimal effort. Though the commonly available, room temperature curing Araldite 2011 epoxy (Huntsman Advanced Materials GmbH, Basel, Switzerland), resulted in rigid structures on the FR4 substrate, the $T_{g}$ of the cured structure was not sufficient to withstand the temperature applied during the wirebonding process. On the other hand, thermal curing two component epoxies AW4510 + HW4510 and AW4804 + HW4804 from Huntsman Advanced Materials have a high $T_{g}$ once cured, however, they showed poor adhesion to the FR4 substrate. Hence an unconventional mixture of AW4510 + HW4804 was prepared. The result was promising since the adhesion to the substrate and the $T_{g}$ were improved. The optimal curing conditions reported in Table 3 were found by trial and error. The automated wirebonding process showed that the final structures made with this new epoxy mixture were stable up to 150 ${}^{{}^{\circ}}$C.

## 3. Chip Design

In addition to the material choice, the geometrical design and the substrate material of the chip also affects the inductive device performance. Generally, the core shape will be straight cylindrical (open-loop), or closed-loop (square, rectangular, or toroidal) to fully guide the magnetic flux. Our solenoidal 3-D coil approach thereby allows to minimize the footprint of the chip without compromising the electrical performance.

The dimension of the PMC core determines the geometry of the solenoid coil that influences the inductance of the coil, thereby the frequency dependent properties of the component.

The self-demagnetization of a magnetic core is another important factor, because it strongly influences the properties and behavior of the core irrespective of the material that it is made of. It increases with the increasing aspect ratio of an open-loop core. Therefore, the core height was chosen to be 2 mm in order to minimize the self-demagnetization for our cores with diameter of 1 mm, *i.e.*, a footprint of 0.79 mm${}^{2}$. The diameter of 1 mm is the least diameter that our casting method could achieve. Also, having post of higher than 2 mm is not applicable as it interferes with the wirebonding fabrication technique. The demagnetizing factor ($N_{z}$) of a cylindrical core having an aspect ratio of 0.5 is 0.18 (in the *z* direction) and is given by the Equation (1) [33]:

(1) $$N_{z}=\frac{1}{2\frac{2n}{\sqrt{\pi }}+1}$$

where *n* is the ratio of diameter and height, *i.e.*, the aspect ratio.

**Figure 2:**
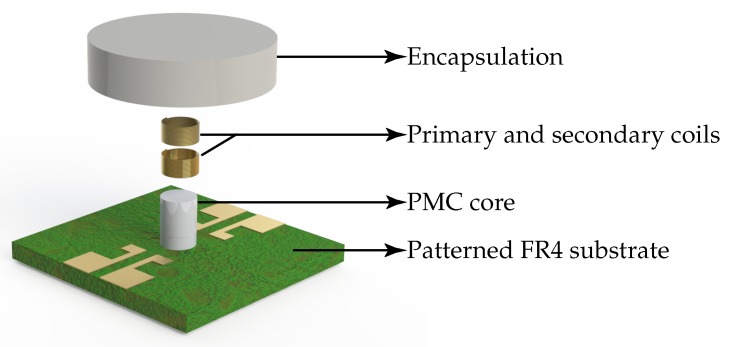
Exploded view of an encapsulated microtransformer chip.

Later, after the coil winding, the core can be encapsulated with the same magnetic composite material as shown in Figure 2. The encapsulation provides self shielding, mechanical stability, and eases the handling of the chips. As a substrate we chose a 0.5 mm thick FR4 from Bungard Elektronik GmbH (Windeck, Germany). This material is expected to have a low substrate induced losses compared, for example, to silicon due to the high insulation of the substrate material. It also provides optimal temperature resistance, good adhesion to mostly available epoxies, and sufficient stiffness for the automated wirebonding.

## 4. Fabrication

**Figure 3:**
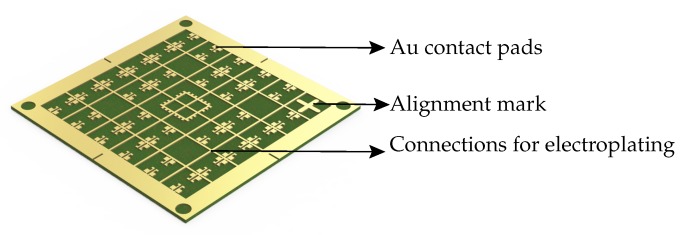
Schematic of the Au electroplated substrate.

**Figure 4:**
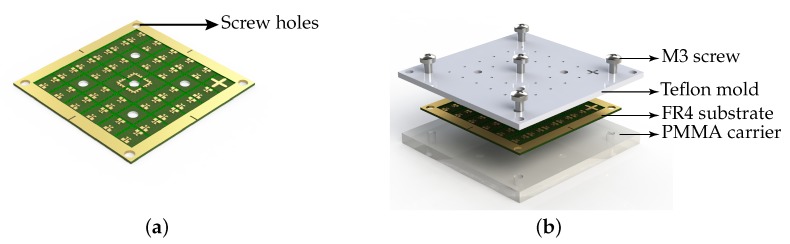
Schematic of the substrate during each fabrication process steps. (**a**) Substrate with contact pads after CNC milling; (**b**) An exploded view of the clamping assembly.

The finalized fabrication process involved simple and cost effective steps. No cleanroom processes were needed, neither for the fabrication of the polymer magnetic cores nor for the fabrication of the inductive coils. The process involved patterning of the 500 µm thick FR4 substrate (dimension: 50 mm × 50 mm) having a 35 µm thick copper layer. Subsequently, the substrate had to be electroplated with 3 µm Au. A 1 µm thick Ni layer was deposited as an intermediate layer to improve the adhesion between copper and gold. The schematic of the Au patterned plated substrate is shown in Figure 3. Then, to disconnect the conductive tracks between the pads which were necessary for the electroplating and to support the final dicing of the fabricated chips, trenches were cut into the substrate using a CNC-mill to a depth of 100 µm as shown in Figure 4a.

The next step was to prepare a mold and clamping carrier for the casting process. Teflon was chosen as the mold material because of its low surface energy due to which it shows low adhesion to most epoxies and polymers. In this work 2 mm thick PTFE sheets from Polymer-Akzent GmbH were used to fabricate the teflon mold. For the clamping carrier 4 mm thick PMMA was chosen in order to allow for visual alignment of the mold and the FR4 substrate. A CNC machine (4030, ISEL) was used to structure both the teflon mold and the PMMA carrier. Once the mold and the carrier were screwed together, the PMC posts could be cast into the clamped substrate and cured. The exploded view of the clamped assembly to cast the PMC is shown in Figure 4b.

**Figure 5:**
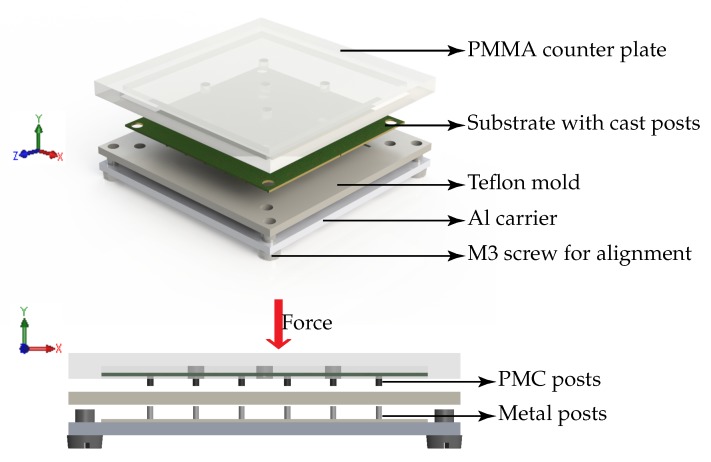
Schematic of the demolding method utilizing the PMMA press to distribute the force along the substrate.

The demolding of the PMC posts from the substrate was one of the crucial steps since the posts could be damaged. Thus, the demolding was facilitated by an additional plate with metal posts and a PMMA counter plate. When aligned with the mold they are used to push the PMC posts out of the mold without any damage as depicted in the Figure 5. Once the PMC posts were made, the following steps were to wind the solenoid coil using an automated wirebonder and then to encapsulate the device.

The wirebonding process was done using a WB3100 (ESEC, Cham, Switzerland) wirebonder. Within 10 s , each coil was wound with up to 60 turns of insulated 25 µm thick gold wire. This winding technique was already well established in the previous works in our laboratory [11,13,16]. However, the bonding parameters such as temperature, ultra-sound power, and pre and process forces for both wedge and ball bonds were determined. These parameters provided successful bonding on the 500 µm thick FR4 substrate.

**Figure 6:**
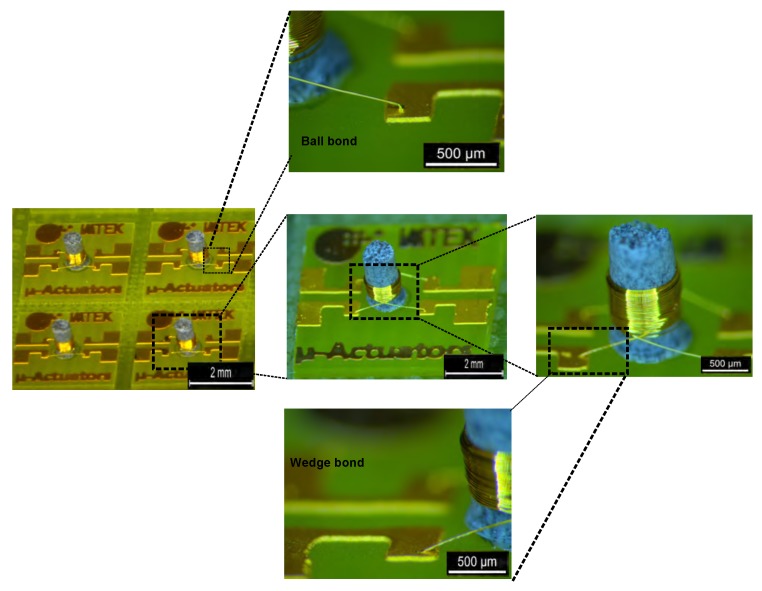
Microscopic images of the magnetic core transformer.Microscopic image of the magnetic core transformer.

PMC cores made out of a mixture of 45%–65% CMD5005 powder with an Araldite AW4510 + HW4804 epoxy were fabricated. The curing conditions for various compositions are listed in the Table 3. Inductor and transformer chips were made with coils having varying number of turns wound on the PMC cores. A microscopic image of the transformers is shown in Figure 6.

**Figure 7:**
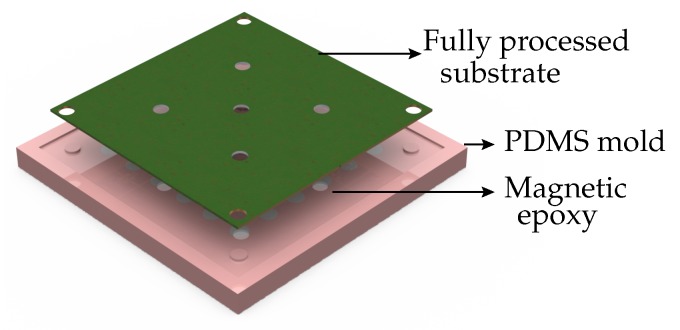
An exploded view of the encapsulation process.

The last and optional process step is the encapsulation of the chips. The encapsulation involved preparation of a PDMS mold in which the encapsulation material was cast, as shown in Figure 7.

The immersed wirebonded posts were cured at 100 ${}^{{}^{\circ}}$C for one hour on a hot plate. Once cured, the PDMS mold was peeled off. The encapsulation material can be chosen for the required performance. For instance, if a magnetic composite is used as encapsulation material, the inductive chip has a higher inductance density than the chip with a non-magnetic encapsulation. However, it compromises the frequency of operation and the electrical performance, due to the addition of a dielectric material and the core induced parasitic losses.

**Figure 8:**
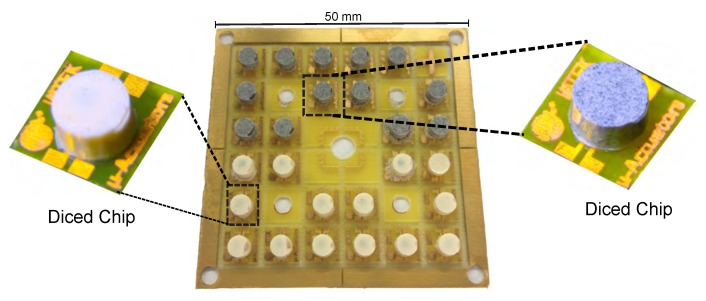
Fully processed substrate with 14 pure epoxy encapsulated transformer chips (white) and 13 magnetic epoxy encapsulated transformer chips (grey).

The chips were diced by using a conventional scissor. The milling step which was done prior to the casting process ensures the ease of the dicing step without any damage to the chips. A fully processed substrate having chips with magnetic as well as non-magnetic encapsulation is shown in Figure 8.

## 5. Measurements and Results

The inductance of a solenoidal coil wound around a magnetic core is directly proportional to the magnetic permeability of the core. Typically, by increasing the magnetic powder percentage the relative magnetic permeability of the PMC core can be increased. However, the highest permeability does not necessarily lead to an optimal performance at the VHF regime [34]. In the PMC cores, two kinds of eddy current losses can be identified, one due to the current circulating within the insulated magnetic particles and the other due to the current around the clusters of particles. With an increasing powder percentage the cluster formation becomes more likely which does not only enhance electrical losses but, besides, will also affect the mechanical stability of the cores. Therefore, the expected optimal percentage to attain the optimal permeability of magnetic powder in the composite has to be determined.

### 5.1. Mechanical Characterization of PMC Cores

**Figure 9:**
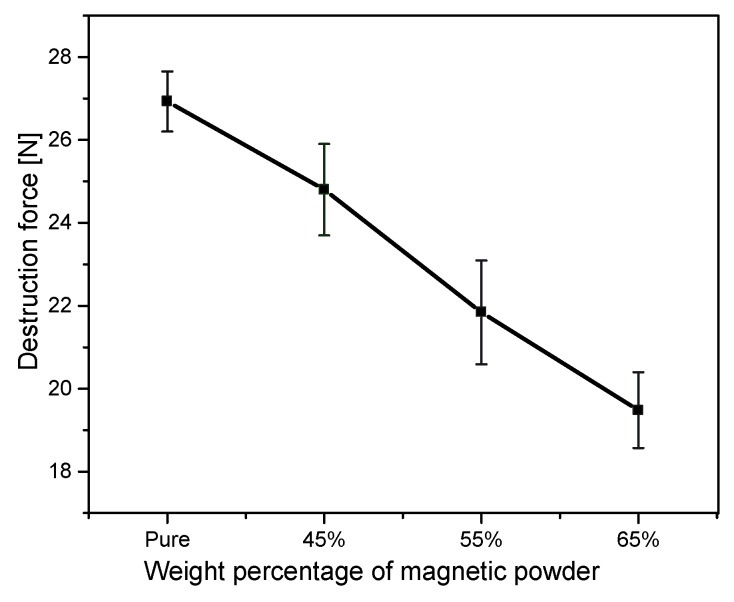
Mechanical stability of the AW4510 based PMC sample posts fabricated on an FR4 substrate. The error bars denote the standard deviation of 5 posts.

The mechanical strength was evaluated using a multipurpose bond tester (Dage 4000, Nordson, Buckinghamshire, UK) to characterize the adhesion of the composite cores to the FR4 substrate. The results of the measurement depicted in Figure 9 show the degradation of the adhesion of the posts to the FR4 substrate with respect to the weight percentage of the magnetic powder in the core composite. The destruction force is the force required to tear of the posts from the substrate at a room temperature of 25 ${}^{{}^{\circ}}$C. For the samples made with a percentage higher than 75% the posts showed poor stability.

### 5.2. Electrical Characterization

The high frequency characterization was performed using an impedance analyzer (E4991A, Agilent Technologies, Santa Clara, CA, USA) in the case of the inductor chips or a vector network analyzer (E5071A, Agilent Technologies) in the case of the transformer chips. A probe-station (9000, Cascade Microtech, Beaverton, OR, USA), equipped with two microprobes (SG/GS-500, Cascade Microtech) was used for the mounting and the measurement. Prior to the measurements, open, short, load, and through (OSLT) calibrations were done and verified using an impedance standard substrate (ISS) (106-683, Cascade Microtech). The measurement was done for the frequency range of 1 MHz to 1 GHz having 801 measurement points.

#### 5.2.1. Inductor Characteristic

The purpose of the inductor characterization was to analyze the influence of the PMC cores and to determine the optimal percentage of the magnetic powder in the composite to achieve both a high inductance and a highest possible quality factor at the VHF frequency range. By extracting the real and the imaginary parts of the impedance, the inductance, electrical resistance, and quality factor of the coils were determined by using the following equations:

(2) $$\begin{array}{c} L=\frac{X_{L}}{2\pi f}=\frac{ℑ\;[Z]}{2\pi f} \end{array}$$

(3) $$\begin{array}{c} R_{e}=ℜ\;\left[Z\right] \end{array}$$

(4) $$\begin{array}{c} Q=\frac{X_{L}}{R_{e}}=\frac{2\pi fL}{R_{e}}=\frac{Imag\;[Z]}{Real\;[Z]} \end{array}$$

**Figure 10:**
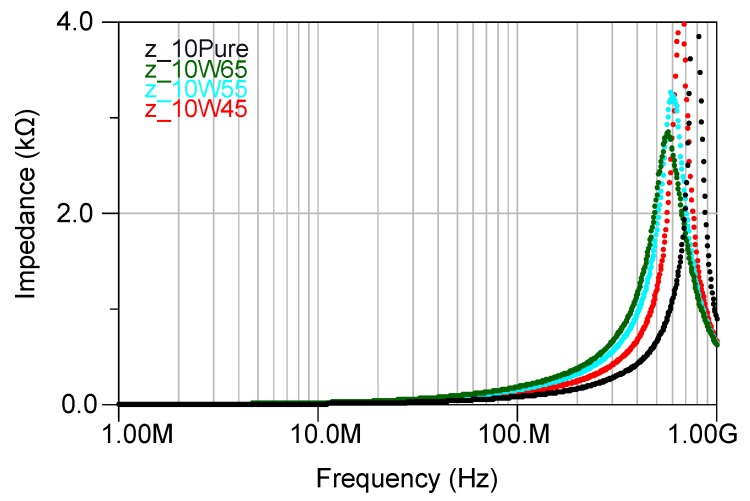
Impedance of the inductors with 10 turns having cores with different percentage of the magnetic powder. Z_10 pure is the inductor with a core made out of the pure epoxy, Z_ 10WY is the impedance of inductors having a core made out of Y% of the magnetic powder mixed with the epoxy.

**Figure 11:**
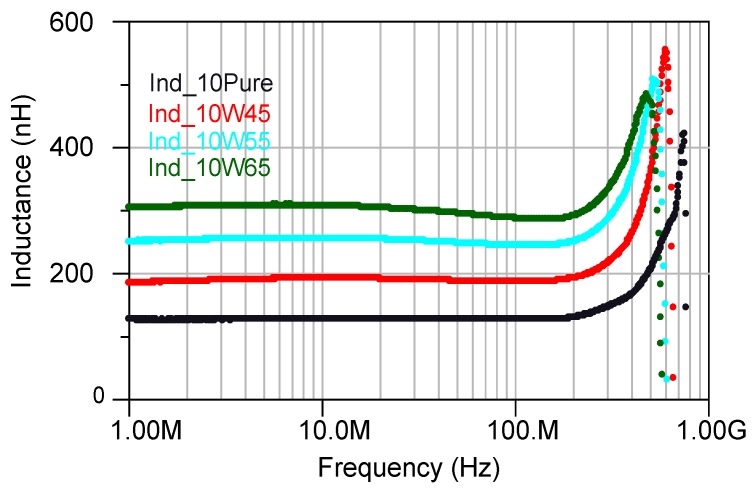
Inductance of the inductors with 10 turns having cores with different percentage of the magnetic powder Ind_10 pure is represents the inductors with pure epoxy core, Ind_XWY stands for inductors having a solenoid made of X turns and core made out of Y% magnetic powder mixed with the epoxy.

Impedance of the inductors with 10 turns having cores with a different percentage of the magnetic powder have been depicted in Figure 10. For the inductors with a higher percentage of the magnetic powder, the impedance started to increase at a lower frequency due to the added core losses in the inductors. Furthermore, the inductance increased with increasing percentage of the magnetic powder as it is shown in Figure 11. However, increasing the inductance caused the self resonance frequency (SRF) to decrease (Figure 10), as described through:

(5) $$SRF=\frac{1}{2\pi \sqrt{L\cdot C_{p}}}$$

where $C_{p}$ is the parasitic capacitance.

**Figure 12:**
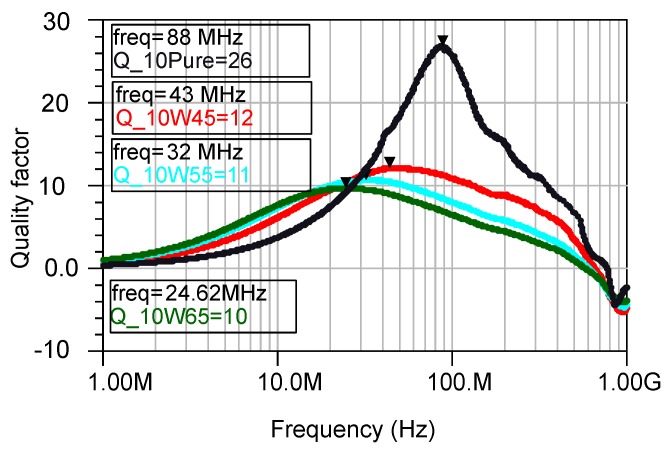
Quality factor of the inductors with 10 turns having cores with different percentage of the magnetic powder. The peak values of each sample and the corresponding frequency are placed in the boxes.

The presence of the magnetic core introduces new losses that compromises the quality factor as in obvious from Figure 12, where the cores with 45% magnetic powder show a slightly higher quality factor than the core with 65%. 

**Figure 13:**
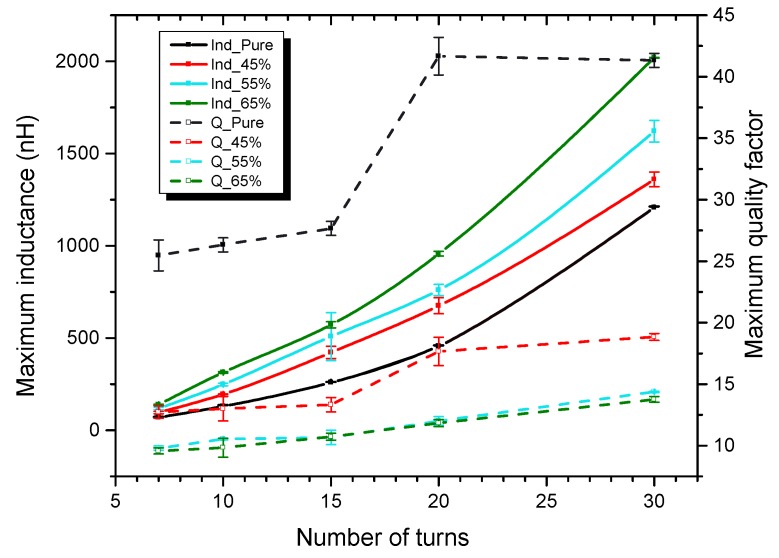
Maximum inductance and quality factor as a function of the number of turns for varying percentage of magnetic powder. The dotted lines indicate the $Q_{max}$ and the solid line the $Ind_{max}$.

In order to study the influence of the number of turns, coils with a varying number of turns (7, 10, 15, 20, 30) were wound on the posts made out of different magnetic composites. Figure 13 shows the measured maximum inductance ($Ind_{max}$) and quality factor ($Q_{max}$) as a function of the number of turns for varying percentage of magnetic powder. The error bars show the deviations of 5 measured similar chips. From this graph we can visualize the trade off between the inductance and quality factor which guides us to a better choice of inductor.

Both the maximum inductance ($Ind_{max}$) and maximum quality factor ($Q_{max}$) increase with the increase in number of turns. However, the DC resistance inherently increases with the increase in number of turns and also affects the $Q_{max}$. This can be visualized by considering the ($Q_{pure}$) value data points at 20 and 30 number of turns. The inductor with 65% magnetic powder provides higher $Ind_{max}$ than the core with 55% magnetic powder at a cost of a slightly smaller $Q_{max}$. Further more the effective permeability of the core can be considered. The inductance of a coil is only a function of its geometry and the permeability of the core and its surrounding medium [16]. For a fixed number of turns, both the pure epoxy core and the magnetic core coils are geometrically identical, hence, we can consider the inductance ratio of a PMC core to the pure epoxy core as the effective permeability ${µ}_{e}$ of the PMC core. The PMC cores with 65% magnetic powder thus showed the highest effective permeability value of 2.4 among all the other PMC cores.

**Figure 14:**
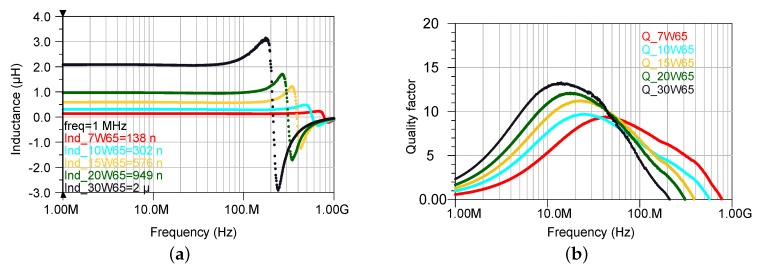
Frequency dependent values of the PMC core inductors for varying number of turns. The composite was made out of 65% powder. (**a**) Inductance; (**b**) Quality factor.

The graphs shown in Figure 14 show the achieved inductance and the quality factor of the PMC cores with 65% magnetic powder for the measured frequency range . The maximum inductance of about 2 µH is achieved for the sample having 30 turns. It shows a constant inductance with a rapid increase near the resonance occurring at 200 MHz. The maximum quality factor of the inductor is 13 at 14 MHz. The values which were achieved here are compared to the previously reported wirebonded microcoils which are listed in Table 4.

#### 5.2.2. Transformer Characteristics

The next objective was to implement transformer chips using coils on PMC cores with 65% magnetic powder and to study the effect of encapsulation with a magnetic epoxy. The flux linkage in a transformer can be defined as a fraction of the total possible flux linkage between the coils. This fractional value is called the coefficient of coupling or coupling factor and is represented by the letter κ, generally expressed as a fractional number between 0 and 1 or a percentage (%) value. It can be calculated from the impedance values by the following equation:

(6) $$\kappa (\%)=100\cdot \sqrt{\frac{Imag\left[Z_{12}\right]\cdot Imag\left[Z_{21}\right]}{Imag\left[Z_{11}\right]\cdot Imag\left[Z_{22}\right]}}$$

The efficiency (η) of a transformer is given by the ratio between the output power and input power. It can be calculated directly from the S-Parameters by using the following equation [36]:

(7) $$\eta =\frac{{\left|S_{21}\right|}^{2}}{1-{\left|S_{11}\right|}^{2}}$$

**Figure 15:**
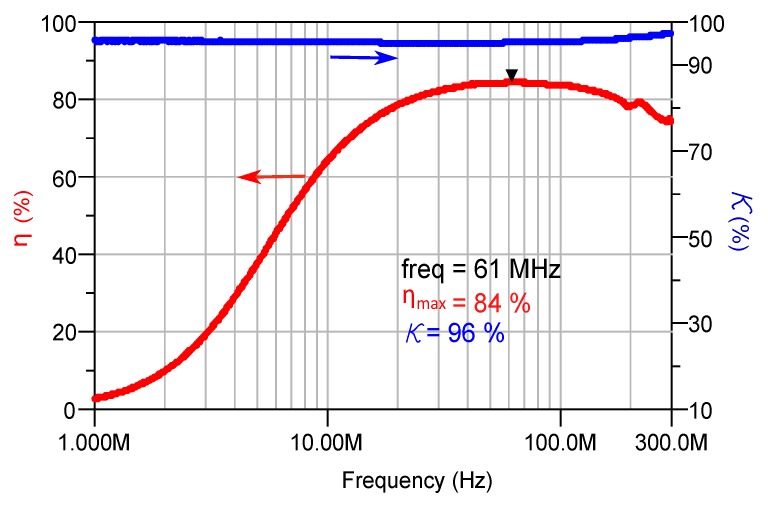
Efficiency and coupling factor of the transformer with PMC core. The sample was made with 10 turns and a turn ratio of 1:1. The composite was made out of 65% powder.

A transformer with 10 turns having a turn ratio of 1:1, wirebonded on PMC cores made out of 65% magnetic composite, was characterized. The measured frequency dependent coupling factor and efficiency of the transformer are shown in Figure 15.

The transformer showed inductance values of 277 nH (primary coil) and 270 nH (secondary coil), and the self resonance frequency was at 419 MHz. The coupling capacitance between the primary and secondary windings of the transformer was 2.35 pF. The measured coupling factor was 96%. Therefore, the magnetic inductance L×κ as well as the leakage inductance L×(1−κ) for the primary coil of the transformer were calculated to be 266 nH and 11 nH, respectively. The efficiency increases with increasing frequency until the losses in the conductor become dominant. The maximum efficiency reached a new value of 84% at 61 MHz. Table 5 proves the high performance of the PMC core microtransformers in terms of the coupling factor and efficiency as well as the small geometrical size of the chips. All the devices except the ones which marked with (*) were characterized using 50 Ω load.

### 5.3. Effect of Encapsulation

**Figure 16:**
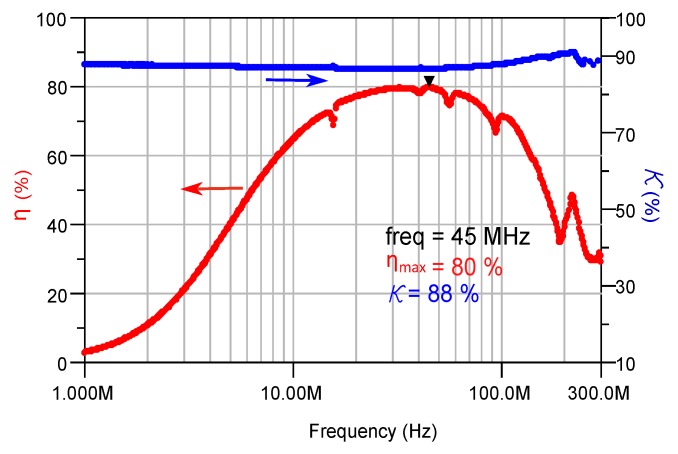
Efficiency and coupling factor of the encapsulated transformer with PMC core. The sample was made with 10 turns and a turn ratio of 1:1 . The core composite was made out of 65% powder. The encapsulation composite was made of 20% powder.

The fabricated transformers with 10 turns were encapsulated with the magnetic epoxy in order to visualize the effect of encapsulation. The magnetic encapsulating epoxy was made out of AW4510 based epoxy mixed with 20% of the NiFeZn magnetic powder. The measured frequency dependent coupling factor and efficiency of the encapsulated transformer is shown in Figure 16. As expected, introducing a dielectric material into the inductive component decreased the self resonant frequency. However, the inductance was increased by a factor of 1.16 since the solenoid is surrounded by a magnetic material. The transformer’s maximum efficiency dropped down to 80% at 45 MHz due to the increase in the parasitic losses.

## 6. Conclusions

An approach for the fabrication of polymer based magnetic core inductive components was developed and presented. This approach utilizes the fully automated wirebonding technique to fabricate the 3-D solenoidal coils. Though the technique was used in our previously published works, the coils were wound on either air cores or laminated magnetic cores before. The open-loop inductor (Ind_20W65) made with an optimized PMC core having 65% magnetic powder reported in this work showed about twice the inductance (960 nH) of the former air core in the VHF regime while having a constant inductance up to 300 MHz, thereby, bridging the gap between the air core and magnetic core devices. Further, by fabricating and characterizing PMC core based transformers, it was verified that the PMC cores also strongly enhance the transformer efficiency and slightly the coupling factor. A transformer made out of PMC having 65% magnetic powder and solenoids with 10 turns (turns ratio 1:1) showed a maximum efficiency of 84% and a coupling factor of 96%.

The developed approach has the ability to fabricate cores with different magnetic powders and various dimensions that could eventually result in the potential to attain even better results. A process for encapsulating the device was also developed and the effect of encapsulation was studied. The complete fabrication process uses well established process like molding and wirebonding. Therefore it is cost effective and easily realizable in a laboratory environment and should be easily transferable to industrial fabrication scale. The passive components in this paper were not designed to be used in a certain converter or isolator circuit. In that case, not only the component, but the whole circuit needs to be considered to measure the load dependent efficiency as we had reported before [16]. 

## Figures and Tables

**Table 1:** Properties of the NiFeZn Soft Magnetic Powder * [25].

Property	Units	Symbol	Standard Test Condition	Value
Initial Permeability	-	${µ}_{i}$	*f* = 10 kHz; *B* $<1\times 10^{-3}$ T	1600±20%
Saturation Flux Density	T	$B_{sat}$	*H* = 795.7 A/m	0.32
Remanence	T	$B_{r}$	-	0.15
Coercive Force	A/m	$H_{c}$	-	15.9
Loss Factor	$10^{-6}$	tan δ/${µ}_{i}$	*f* = 1 MHz; *B* = 0.1 $\times 10^{-3}$ T	≤250
Temperature Coefficient of ${µ}_{i}$	%/${}^{{}^{\circ}}$C	-	-	≤0.7
Volume Resistivity	Ω·cm	ρ	-	$1\times 10^{8}$
Curie Temperature	${}^{{}^{\circ}}$C	$T_{c}$	-	≥130

* Values are typical and based on measurements of a standard toroid at 25 ${}^{{}^{\circ}}$C.

**Table 2:** Binder polymers for fabricating PMC cores to be used in high frequency applications.

Polymers	Dielectric Constant	Young’s Modulus	Water Absorption	CTE	$T_{g}$
		(GPa)	(ISO 62-80) (%)	(PPM/${}^{{}^{\circ}}$C)	(${}^{{}^{\circ}}$C)
BCB	2.7	2.9	0.24	52	>350
Avatrel	2.5	0.5–1.56	0.06	83	250
LCP	3	40	0.1	16	150
KAPTON *	3.5	2.5	2.5	20	360–410
Su8	3	4.02	0.55	52	210
PDMS	2.8	0.4–0.9	>1	310	-
Epoxy (Araldite)	3	>5	<0.8	<15	50–100

* Deposition of particles on film.

**Table 3:** Process conditions for obtaining the AW4510 + HW4804 based epoxy composite core with 1 mm in diameter and 2 mm height. The pre-heating was done before dispensing the composite to enhance the viscocity of the mixture. The post curing was done at 100 ${}^{{}^{\circ}}$C in an oven for 1 h.

Percentage of Magnetic Powder	Pre- Heating Time (50 ${}^{{}^{\circ}}$C)	Waiting Time before Dispensing
(%)	(min)	(min)
Pure	15	45
45	10	15
55	10	10
65	5	10

**Table 4:** Comparison between the inductance achieved in this work and the previous work.

N_Core Used	*L* @ 1 MHz	$Q_{max}$ @ MHz	SRF
	(nH)		(MHz)
20_Air Core [encapsulated] [15]	464	22 @ 78	218
18_Metglas Core [Open-loop] [35]	1024	9 @ 37	130
40_Metglas Core [Closed-loop] * [16]	$39.9\times 10^{3}$	7 @ 0.3	27
Ind_30_W45	1300	19 @ 43	300
Ind_30_W55	1700	14 @ 30	204
Ind_30_W65	2090	13 @ 14	200
Ind_20_W65	961	12 @ 17	300

* The sample’s coil was made of 25 µm thick insulated copper wire; All other samples were wound with 25 µm insulated gold wire.

**Table 5:** Survey on the state of the art microtransformers (pure RF microinductors are excluded). All the devices except the ones which marked with (*) were characterized using 50 Ω load.

Refferences	Citation	L/$R_{DC}$	$\eta_{max}$	$F_{\eta \;max}$	κ	Core Volume	Layout	Core Material
		(nH/Ω)	(%)	(MHz)	(%)	(mm${}^{3}$)		
Rassel *et al*., 2003	[37]	100	10 ${}^{*}$	0.5	90	0.025	Solenoid	NiFe
Yamaguchi *et al*., 1993	[38]	357	29 ${}^{*}$	20	90	3.77	Planar	CoFeSiB
Allen *et al*., 2003	[39]	371	32	26	85	3.99	Planar	NiFe
Meyer *et al*., 2010	[40]	52.6	40	50	63	-	Stack planar	Air
Brunet *et al*., 2002	[41]	900	40 ${}^{*}$	2	-	1.45	Planar	NiFe
Xu *et al*., 1998	[42]	720	45	10	90	0.520	Solenoid	NiFe
Sakakibara *et al*., 1996	[43]	106	50	10	93	10	Planar	CoZrRe
Kurata *et al*., 1994	[44]	24	50	100	92	0.042	Solenoid	CoFeSiB
Tang *et al*., 2001	[45]	154	63	8	95	-	Planar	Air
Wang *et al*., 2007	[8]	840	63	10	93	0.355	Planar	NiFe
Raimann *et al*., 2012	[11]	142.3	65	45	86	1.6	Solenoid	Ferrite
Moazenzadeh *et al*., 2013	[15]	175.6	68	54	94	0.674	Solenoid	Air
Moazenzadeh *et al*., 2014	[16]	5714	71	1.11	98	7.5	Solenoid	CoFeNiSiB
Moazenzadeh *et al*., 2013	[46]	395	74	32	98	0.720	Solenoid	CoFeNiSiB
Wu *et al*., 2015	[47]	195.5	-	-	98	0.2	Planar	Air
Macrelli *et al*., 2014	[48]	73256	-	-	90	4.32	Toroid	MnZn
Khan *et al*., 2015	[49]	16.2	∼55	-	75	0.044	Fractal shaped	Air
Tra_10_W65 *	This work	158	**84**	61	**96**	**1.6**	Solenoid	NiFeZn+epoxy

* Tra_X_WY represents the transformer made of X number of turns in primary and secondary coil wound around the composite core made of Y% of magnetic powder.
