# Correction: Mariappan, S.G. *et al*. Polymer Magnetic Composite Core Based Microcoils and Microtransformers for Very High Frequency Power Applications. *Micromachines*, 2016, *7*, 60

**DOI:** 10.3390/mi7060100

**Published:** 2016-06-08

**Authors:** Saravana Guru Mariappan, Ali Moazenzadeh, Ulrike Wallrabe

**Affiliations:** 1 Laboratory for Microactuators, Department of Microsystems Engineering (IMTEK), University of Freiburg, Georges-Koehler-Allee 102, 79110 Freiburg, Germany; guru.mariappan@voxalytic.com (S.G.M.); ali.moazenzadeh@voxalytic.com (A.M.); 2 Voxalytic GmbH, Rosengarten 3, 76228 Karlsruhe, Germany

In the published paper [1], there are two errors in Table 5, both of which correspond to the reported results of a paper authored by Macrelli *et al.* in 2014 [48]. The correct value of the inductance per DC resistance of this paper is 73256 nH/Ω, and the correct value of the core volume is 4.32 mm${}^{3}$. Therefore, Table 5 should read as follows:

The authors apologize for any inconvenience caused by these errors. The manuscript will be updated online and the previous version will remain available on the article webpage.

## Figures and Tables

**Table 5:** Survey on the state of the art microtransformers (pure RF microinductors are excluded). All the devices except the ones which marked with (*) were characterized using 50 Ω load.

Refferences	Citation	L/$R_{DC}$	$\eta_{max}$	$F_{\eta \;max}$	κ	Core Volume	Layout	Core Material
		(nH/Ω)	(%)	(MHz)	(%)	(mm${}^{3}$)				
Rassel *et al*., 2003	[37]	100	10 ${}^{*}$	0.5	90	0.025	Solenoid	NiFe
Yamaguchi *et al*., 1993	[38]	357	29 ${}^{*}$	20	90	3.77	Planar	CoFeSiB
Allen *et al*., 2003	[39]	371	32	26	85	3.99	Planar	NiFe
Meyer *et al*., 2010	[40]	52.6	40	50	63	-	Stack planar	Air
Brunet *et al*., 2002	[41]	900	40 ${}^{*}$	2	-	1.45	Planar	NiFe
Xu *et al*., 1998	[42]	720	45	10	90	0.520	Solenoid	NiFe
Sakakibara *et al*., 1996	[43]	106	50	10	93	10	Planar	CoZrRe
Kurata *et al*., 1994	[44]	24	50	100	92	0.042	Solenoid	CoFeSiB
Tang *et al*., 2001	[45]	154	63	8	95	-	Planar	Air
Wang *et al*., 2007	[8]	840	63	10	93	0.355	Planar	NiFe
Raimann *et al*., 2012	[11]	142.3	65	45	86	1.6	Solenoid	Ferrite
Moazenzadeh *et al*., 2013	[15]	175.6	68	54	94	0.674	Solenoid	Air
Moazenzadeh *et al*., 2014	[16]	5714	71	1.11	98	7.5	Solenoid	CoFeNiSiB
Moazenzadeh *et al*., 2013	[46]	395	74	32	98	0.720	Solenoid	CoFeNiSiB
Wu *et al*., 2015	[47]	195.5	-	-	98	0.2	Planar	Air
Macrelli *et al*., 2014	[48]	73256	-	-	90	4.32	Toroid	MnZn
Khan *et al*., 2015	[49]	16.2	∼55	-	75	0.044	Fractal shaped	Air
Tra_10_W65 *	This work	158	**84**	61	**96**	**1.6**	Solenoid	NiFeZn+epoxy

* Tra_X_WY represents the transformer made of X number of turns in primary and secondary coil wound around the composite core made of Y% of magnetic powder.
